# *PRRT2* ‐positive self‐limited infantile epilepsy: Initial seizure characteristics and response to sodium channel blockers

**DOI:** 10.1002/epi4.12708

**Published:** 2023-02-20

**Authors:** Jiwon Lee, Young Ok Kim, Byung Chan Lim, Jeehun Lee

**Affiliations:** ^1^ Department of Pediatrics, Samsung Medical Center Sungkyunkwan University School of Medicine Seoul South Korea; ^2^ Department of Pediatrics Chonnam National University Medical School Gwangju South Korea; ^3^ Department of Pediatrics, Seoul National University Children's Hospital Seoul National University College of Medicine Seoul South Korea

**Keywords:** benign partial epilepsy in infancy, genetic infantile epilepsy, PRRT2 gene, self‐limited infantile epilepsy, sodium channel blocker

## Abstract

**Objective:**

Self‐limited infantile epilepsy (SeLIE) has distinctive clinical features, and the *PRRT2* gene is known to be a considerable genetic cause. There have been a few studies on *PRRT2*‐positive SeLIE only, and anti‐seizure medications are often required due to frequent seizures at initial seizure onset. This study aimed to provide clinical information for the early recognition of patients with *PRRT2*‐positive SeLIE and to propose effective anti‐seizure medications for seizure control.

**Methods:**

We retrospectively reviewed 36 patients diagnosed with SeLIE with genetically confirmed pathogenic variants of *PRRT2*. In addition, six atypical cases with neonatal‐onset seizures and unremitting after 3 years of age were included to understand the expanded clinical spectrum of *PRRT2*‐related epilepsy. We analyzed the initial presentation, clinical course, and seizure control response to anti‐seizure medications.

**Results:**

Patients with *PRRT2*‐related epilepsy had characteristic seizure semiology at the initial presentation, including all afebrile, clustered (n = 23, 63.9%), short‐duration (n = 33, 91.7%), and bilateral tonic–clonic seizures (n = 26, 72.2%). Genetic analysis revealed that c. 649dupC was the most common variant, and six patients had a 16p11.2 microdeletion containing the *PRRT2* gene. One‐third of the patients were sporadic cases without a family history of epilepsy or paroxysmal movement disorders. In the 33 patients treated with anti‐seizure medications, sodium channel blockers, such as carbamazepine, were the most effective in seizure control.

**Significance:**

Our results delineated the clinical characteristics of *PRRT2*‐positive SeLIE, differentiating it from other genetic infantile epilepsies and discovered the effective anti‐seizure medications for initial clustered seizure control. If afebrile bilateral tonic–clonic seizures develop in a normally developed infant as a clustered pattern, *PRRT2*‐positive SeLIE should be considered as a possible diagnosis, and sodium channel blockers should be administered as the first medication for seizure control.

**Key Points**

- *PRRT2*‐positive self‐limited infantile epilepsy (SeLIE) has clinical features distinguishing it from other types of infantile epilepsy at the initial presentation.
- The initial seizures of *PRRT2*‐positive SeLIE were afebrile bilateral tonic–clonic seizures with a short duration, occurring as a clustered pattern.
- Sodium channel blockers have shown promising efficacy in controlling frequent seizures in patients with *PRRT2*‐positive SeLIE.

## 1 INTRODUCTION

The incidence of epilepsy is high in the first year of life. The clinical presentation and course of early‐onset epilepsy syndromes are diverse and include both self‐limited epilepsy syndromes and developmental epileptic encephalopathies.^1^ Self‐limited infantile epilepsy (SeLIE), previously termed benign partial epilepsy in infancy, was shown to have the highest incidence among self‐limited epilepsies during early childhood.^2^

Self‐limited infantile epilepsy is distinctly described in terms of seizure onset, seizure type, and course of illness. The pathogenic variants in *PRRT2* have been found in up to 70% of SeLIE patients.^2^, ^3^, ^4^ Since most studies have included *PRRT2*‐positive and *PRRT2*‐negative patients as a group for SeLIE, there is a need to precisely characterize patients with *PRRT2‐*positive SeLIE. The seizure onset of SeLIE frequently overlaps with those of other infantile‐onset genetic epilepsy syndromes.^3^ Despite the natural course of resolution by 2 years of age in most cases, patients often require anti‐seizure medications due to frequent and clustered seizures at the initial presentation. An early genetic diagnosis of *PRRT2*‐positive SeLIE can partly predict the clinical course from the onset, including the response to anti‐seizure medications. Recent studies reporting the effectiveness of sodium channel blockers (SCB) in patients with SeLIE are illustrative examples.^5^, ^6^ However, since it usually takes months to obtain genetic testing results, this has also limited clinical utility when deciding on anti‐seizure medications at the time of seizure onset. Thus, the electroclinical features at seizure onset in patients with *PRRT2*‐positive SeLIE need to be specifically investigated.

Based on these considerations, we reviewed the clinical characteristics and course of patients diagnosed with SeLIE confirmed to have pathogenic variants of the *PRRT2* gene. The patient's group also included some patients with neonatal‐onset seizures and those with unremitting seizures, even after 3 years of age, to investigate the extended spectrum of *PRRT2*‐related epilepsy. Our study findings can aid in identifying patients with *PRRT2*‐related epilepsy at an early stage of the disease and suggest a suitable first‐line anti‐seizure medication.

## 2 METHODS

### 2.1 Patients and genetic evaluation

The patients were enrolled in three tertiary hospitals in Korea between January 2012 and July 2022. They met the following inclusion criteria: patients had their first seizure developing before 1 year of age without provocative factors, normal developmental milestones before seizure onset, no abnormal findings on brain magnetic resonance imaging (MRI) that could be the cause of their seizures, and confirmed pathogenic sequence or structural variants in the *PRRT2* gene.

The pathogenicity of *PRRT2* variants was classified based on the international guidelines proposed by the American College of Medical Genetics (ACMG) and the Association for Molecular Pathology (AMP).^7^ The detected variants were confirmed using Sanger sequencing. In the case of *PRRT2* deletion, copy number variation was analyzed using a chromosomal microarray. Clinical and laboratory data were reviewed retrospectively for all patients, including the age of symptom onset, family history of seizure and paroxysmal movement disorders, semiology of the seizure, administration of medications, response to initial anti‐seizure medication (ineffectiveness was defined as recurrence of seizure requiring additional anti‐seizure medication), electroencephalography (EEG) results, and brain MRI findings. Seizure type was classified according to the operational classification proposed by International League Against Epilepsy.^8^ Since focal onset features could not be determined based on the caregiver's description, we did not use either generalized or focal for the classification of bilateral tonic–clonic seizures. Clustered seizure was defined as seizure recurrence within 24 hours. Anti‐seizure medications were divided into SCB and non‐SCB, and SCB included carbamazepine, oxcarbazepine, and phenytoin; non‐SCB included levetiracetam, phenobarbital, valproic acid, zonisamide, and clobazam.

## 3 RESULTS

### 3.1 Demographic features and genetic variants

Thirty‐six patients (20 females and 16 males) diagnosed with *PRRT2*‐related epilepsy were included in this study. Six atypical cases were included to expand our understanding of the clinical spectrum of *PRRT2*‐related epilepsy: three patients with neonatal‐onset seizures and three with unremitting seizures after 3 years of age. The mean follow‐up duration was 8.3 ± 4.6 years. Twenty‐two patients (61.1%) had a family history of epilepsy (n = 13, 36.1%), paroxysmal kinesigenic dyskinesia/choreoathetosis (n = 3, 8.3%), and both (n = 6, 16.7%). Fourteen patients (38.9%) were sporadic. None of the patients had delayed development before seizure onset. The initial EEG and brain MRI results were normal in 33 and 32 patients, respectively. One patient had evidence of perinatal cerebral infarction in the left frontal lobe on brain MRI. The other three patients did not undergo brain MRI because of a definite family history of SeLIE or paroxysmal kinesigenic dyskinesia and genetic confirmation of pathogenic variants of the *PRRT2* gene. The most common pathogenic variant of the *PRRT2* gene was c.649dupC (p.Arg217Profs*8), found in 24 patients (66.7%). There were six cases of microdeletions, including the region corresponding to the *PRRT2* gene. The size of the microdeletion was 0.4–0.5 Mbs. Four other variants were identified in six patients as follows: c.640delinsCC (n = 2), c.966delG (n = 2), c.548dupG (n = 1), and c.769_797insGG (n = 1). Detailed clinical features are described in Table S1.

### 3.2 Characteristics of the initial seizure

**FIGURE 1:**
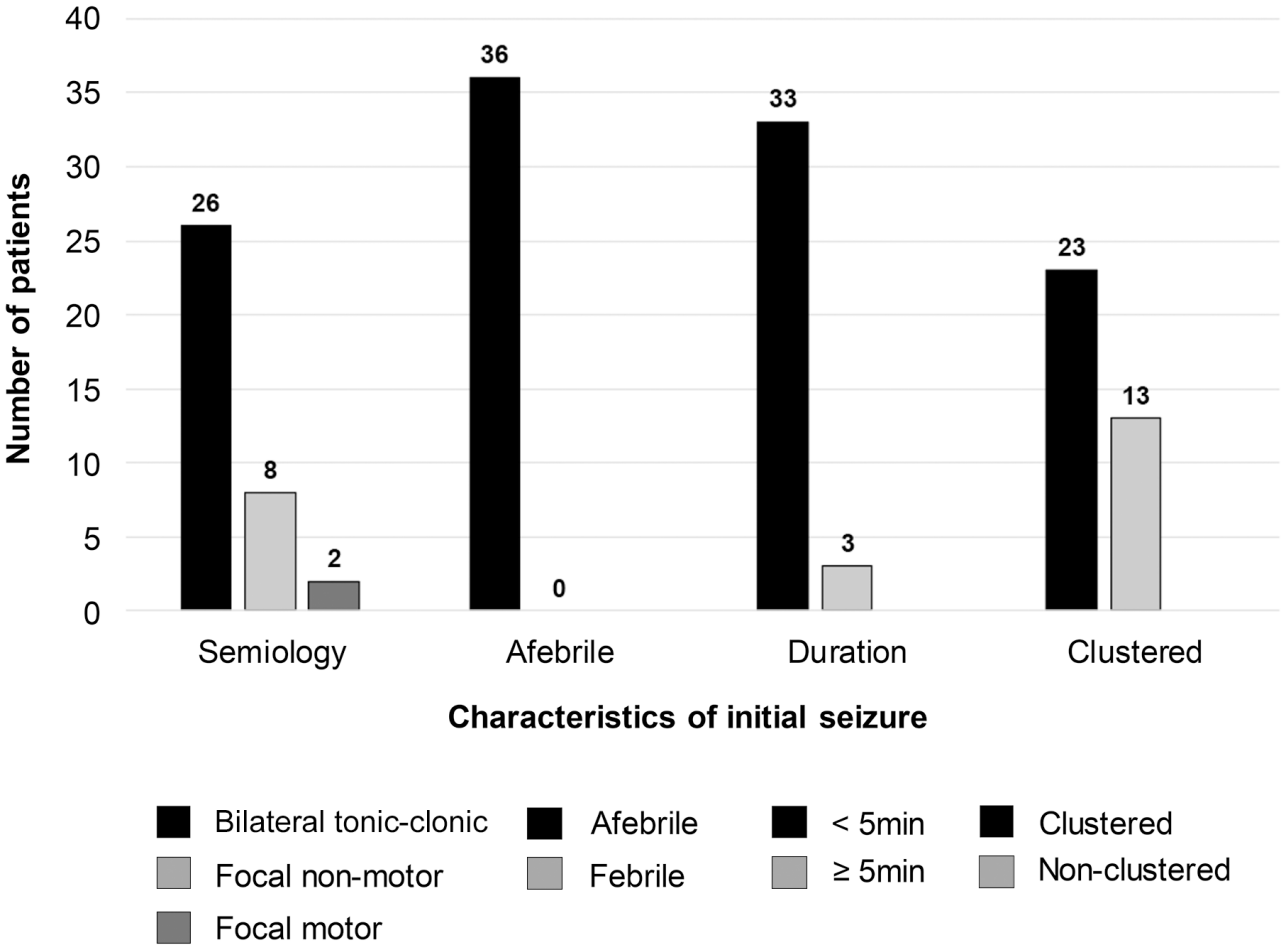
Characteristics of the initial seizures in *PRRT2*‐positive self‐limited infantile epilepsy. Bilateral tonic–clonic seizures were the most common type (n = 26), and focal motor seizures were rare (n = 2) at initial presentation. All initial seizures were afebrile, and 33 patients experienced brief seizures lasting <5 min. In 23 patients (63.9%), the seizures occurred in a clustered pattern.

The mean age at seizure onset was 4.1 ± 1.94 months (range, 3 days – 9 months). Most patients (n = 30, 83.3%) had their first seizure at 2–6 months of age. Three patients (8.3%) experienced their first seizure within 1 week after birth. The most common semiology of the initial seizure was a bilateral tonic–clonic seizure in 72.2% of patients (n = 26), followed by a focal nonmotor seizure with impaired awareness in eight patients. Their first seizures were all afebrile, and most (n = 32) had short seizures of less than 5 minutes. At the initial presentation, 23 patients (63.9%) had clustered seizures. Approximately half of the patients (n = 17) were administered parenteral anti‐seizure medications, such as phenobarbital, levetiracetam, phenytoin, and valproic acid, for frequent seizures. Figure 1 shows the characteristics of the initial seizure in the patients.

### 3.3 Response to the initial anti‐seizure medication

**FIGURE 2:**
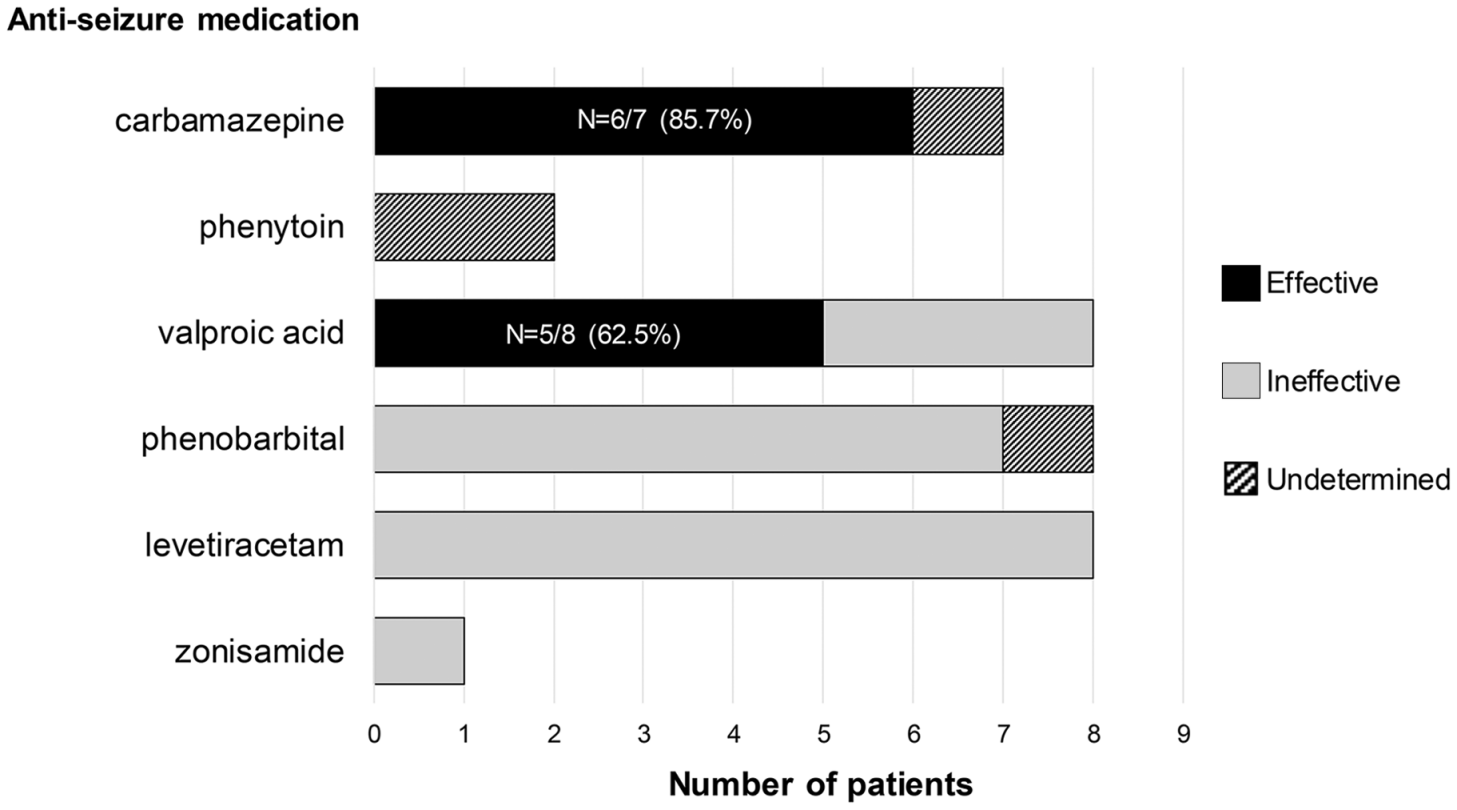
Response of initial seizure control according to anti‐seizure medication. Carbamazepine was the most effective (85.7%) in *PRRT2*‐positive self‐limited infantile epilepsy, followed by valproic acid in five patients (62.5%). Despite being administered to the largest number of patients, levetiracetam and phenobarbital could not initially control seizures. Four patients for whom the anti‐seizure medication effectiveness was undetermined discontinued the medication due to rash (one with carbamazepine) or had poor drug compliance (two with phenytoin and one with phenobarbital).

Among the 33 patients receiving anti‐seizure medications, 20 required a second anti‐seizure medication due to uncontrolled seizures. We divided the patients into two groups according to the type of the first medication: one administered SCB (n = 9) and the other administered non‐SCB (n = 24). Table 1 shows a comparison of clinical responses between the two groups. There was no difference in the age at seizure onset. All patients who were administered SCB as the first medication resolved before 1 year of age (age of the last seizure, 4–9 months), and their seizures were controlled with SCB except for two patients. In the group that was administered non‐SCB as the first medication, 75% of patients (n = 18) required a second anti‐seizure medication for recurrent seizures. The response rate of individual anti‐seizure medications determined by the proportion of patients who did not need an additional anti‐seizure medication is illustrated in Figure 2. While carbamazepine and valproic acid showed higher response rates: 85.7% (n = 6/7) and 62.5% (n = 5/8), respectively, nearly all the patients initially treated with levetiracetam and phenobarbital required additional anti‐seizure medications due to inadequate seizure control.

**TABLE 1:** Comparison of patients taking sodium channel blockers (SCB) and non‐SCB as the first anti‐seizure medication.

	SCB as the first anti‐seizure medication	Non‐SCB as the first anti‐seizure medication
Number of patients	9	24
Onset age (mon)	3.3 ± 1.0	3.0 ± 0.0
Age of last seizure (mon)	5.9 ± 1.7	22.7 ± 43.6
Recurred seizure after 1 y old	none	4
Recurred seizure after 3 y old	none	3
Administration of the second medication	2	18
Reason for the second medication	1—low drug level in blood (phenytoin) 1—stopped due to rash	Inadequate seizure control by the first medication

*Note*: Data are the number of patients. The age represents mean ± standard deviation.

### 3.4 Clinical course

**FIGURE 3:**
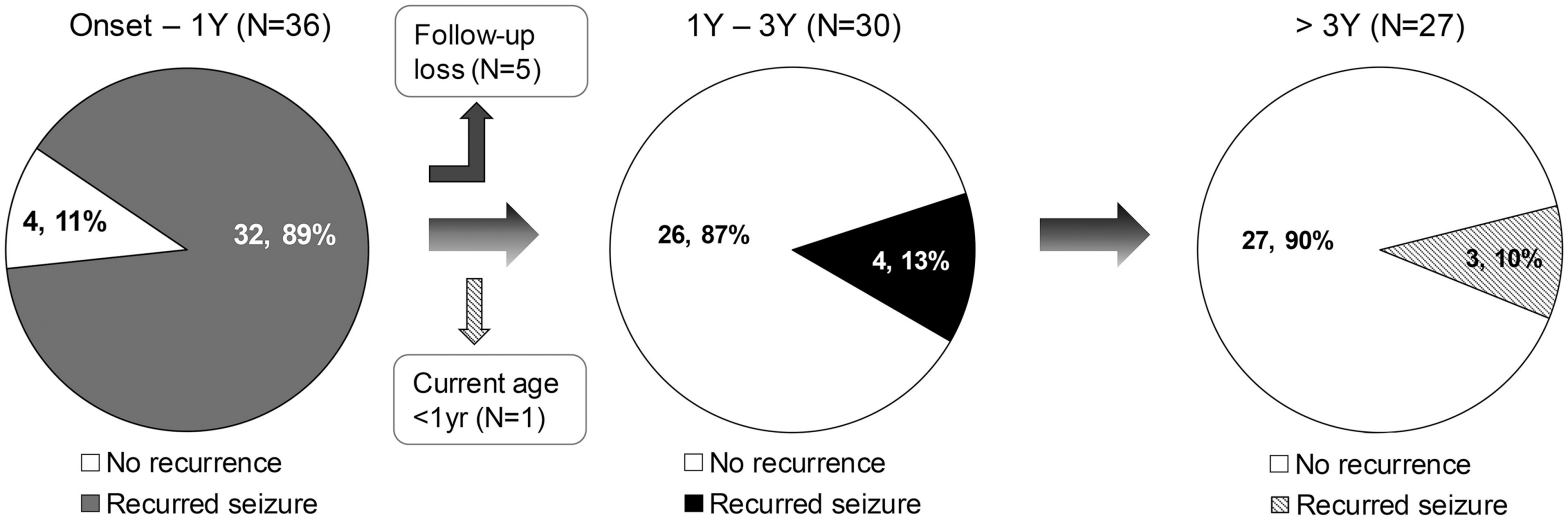
Clinical evolution according to age. Four patients showed only one seizure at initial presentation. The remaining 32 patients had at least two seizures by the age of 1 year. After 1 year of age, five patients were lost to follow‐up. One patient was under 1 year of age and was excluded from follow‐up after 1 year of age. From 1–3 years of age, 26 of the 30 patients had a self‐limited course of seizures. Finally, three patients were treated with anti‐seizure medications for repeated seizures after 3 years of age. Two of these patients had a self‐limited course after 1 year of age, and the other one had repeated seizures until 3 years of age (the one represented by the black section in the second pie chart).

The clinical course of all patients according to age is shown in Figure 3. A total of 33 patients were administered anti‐seizure medications for recurrent seizures after the first seizure onset, except for one. The exception was a girl whose family members (father and older brother) were diagnosed with SeLIE and paroxysmal kinesigenic dyskinesia caused by a *PRRT2* variant and whose parents wanted to prevent the recurrence of seizures until self‐remission. After 12 months of age, 30 patients were followed up, and only four (13%) had recurrent seizures. Among them, three patients became seizure‐free before 3 years of age, but one (No. 03) had recurrent seizures after 3 years of age.

Three of the 36 patients, including the aforementioned patient (No. 03) and two patients who remained seizure‐free between one and 3 years of age but relapsed after 3 years of age, required anti‐seizure medications for repeated seizures after 3 years of age. Patient No. 3 showed an unprovoked focal motor seizure at 10.5 years of age. He had been treated with oxcarbazepine for 3 years since the last seizure, after which it was discontinued as there were no more seizures. One patient (No. 01) of the other two patients had three episodes of seizures before 1 year of age and showed excellent response to valproic acid. At the age of 1 year, he had mild developmental delay. At 5 years of age, bilateral tonic–clonic seizures recurred and were well‐controlled by valproic acid, which initially responded well to treatment. The last patient (No. 08) had seizure recurrence at 7 years, which was revealed to arise from the frontal lobe via video‐EEG monitoring. Phenytoin and carbamazepine were used as initial treatments for his seizures, and even when they recurred, he had no seizures since taking carbamazepine.

Five of the patients who participated in this study developed paroxysmal kinesigenic dyskinesia in their teenage years, but its exact incidence rate cannot be determined since more than half (19 patients) were still under 10 years of age.

## 4 DISCUSSION

This study delineates the clinical characteristics and response to anti‐seizure medications in patients with *PRRT2*‐positive SeLIE, distinguishing them from those of other early‐life epilepsy syndromes. The initial seizures were all afebrile, and most cases were clustered seizures with a short duration. The most common semiology was bilateral tonic–clonic seizure, in 72.2% of patients, and one‐third had no family history of seizures or paroxysmal movement disorders. More than 90% of them required anti‐seizure medications due to the high seizure burden for 1 year after the onset and responded well to SCBs. Therefore, in clinical cases of repeated brief afebrile bilateral tonic–clonic seizures at initial onset, *PRRT2*‐related epilepsy should be considered, and SCBs should be used for seizure control.

There are various genetic causes of SeLIE, and the primary causal gene is known to be *PRRT2*, accounting for two‐thirds of cases. The remaining 30% includes the *SCN8A*, *KCNQ2*, *SCN2A*, and *KCNQ3* genes.^2^, ^9^, ^10^ However, these genes are known to be different in the peak onset of seizures from *PRRT2*‐related epilepsy,^11^, ^12^, ^13^, ^14^ and little is known about the electroclinical features when the seizure onset of patients with pathogenic variants in these genes overlaps with SeLIE. *SCN1A* is linked with Dravet syndrome and is one of the genes whose seizure onset age overlaps with *PRRT2*.^3^ The seizures during infancy in Dravet syndrome have been well characterized as prolonged hemiclonic seizures in a febrile context.^9^, ^15^ However, in approximately one‐third of patients with Dravet syndrome, initial seizures may not be prolonged or occur in an afebrile context.^16^ Our previous study on *SCN1A*‐positive Dravet syndrome patients also revealed that the seizure was prolonged (>15 minutes) in 29% of patients and was associated with fever in 51% of the total patients at onset.^17^ Since developmental problems might not be evident at onset, some clinical features at onset could overlap between *PRRT2*‐positive SeLIE and *SCN1A*‐positive Dravet syndrome. Thus, the differentiating features of *PRRT2*‐positive SeLIE patients at onset need to be characterized more specifically. In the present study, the semiology of the initial seizure was classified as bilateral tonic–clonic in over two‐thirds (72.2%) of the patients, and initial seizures were clustered in 63.9% of the patients. Although all these features were described in earlier clinical studies,^18^, ^19^ they seemed more accentuated when SeLIE patients were restricted to *PRRT2*‐positive patients. In particular, the high frequency of short bilateral tonic–clonic seizures in *PRRT2*‐positive patients could be in sharp contrast to the prolonged hemiclonic seizures in *SCN1A*‐positive patients. Collectively, for differentiation from *SCN1A*‐positive Dravet syndrome at onset, it is noteworthy that there are fewer hemiclonic seizures, and they occur as clustered patterns based on our findings, in addition to the well‐known afebrile and short‐duration seizures.

In *PRRT2*‐related epilepsy, a prompt decision regarding anti‐seizure medication is often required because of clustered seizures at the initial presentation. As indicated in the present study, for rapid seizure control, parenteral administration of anti‐seizure medication was needed in almost half of the patients (47.2%). Furthermore, levetiracetam and valproic acid were preferred over phenytoin as parenteral anti‐seizure medications. This preference might reflect the notion that seizures can often be aggravated by using SCB in *SCN1A*‐positive patients. Although SCB was used as the first anti‐seizure medication in only 27.2% of the treated patients in our study, SCBs did not require the use of a second anti‐seizure medication due to recurrent seizures. Recent studies have also reported a favorable seizure response to lacosamide and oxcarbazepine.^5^, ^6^, ^20^ Lamotrigine has been reported to be effective in patients with paroxysmal kinesigenic dyskinesia caused by the *PRRT2* gene.^21^, ^22^ Considering its favorable side effect profile, lamotrigine could be a promising option as an initial treatment. In a previous study, PRRT2 interacted with voltage‐dependent sodium channels in a murine model and is a significant negative modulator of Nav1.2 and Nav1.6 channels.^23^ This experimental evidence and our clinical study support using SCBs as the first anti‐seizure medication in *PRRT2*‐positive SeLIE.

Our data included atypical cases of neonatal‐onset and unremitting seizures after 3 years of age. The peak age of seizure onset in *PRRT2*‐related epilepsy is three to 9 months.^24^ Neonatal onset of seizures is uncommon, and a few cases have been reported in previous studies.^25^, ^26^ Our data included three patients with neonatal‐onset seizures from three to 7 days of life. Although they had developed seizures at a younger age than others, their course did not differ from that of other patients with infantile‐onset seizures. They had their last seizures before 6 months of age and developed normally. *PRRT2*‐related epilepsy has normal neurodevelopmental milestones and spontaneous remission of seizures before two to 3 years of age, approximately.^27^, ^28^, ^29^ In our study, three patients continued to take anti‐seizure medications for seizures after that age. However, these patients also responded well to SCBs and were seizure‐free. Although these atypical cases could not be classified as SeLIE, they will contribute to the complete understanding of the clinical spectrum of *PRRT2*‐related epilepsy.

In conclusion, our study demonstrated that *PRRT2*‐positive SeLIE has peculiar clinical features at the initial presentation and an early predisposing age of onset. These findings are distinct from those of other genetic epilepsies that develop at a similar age but have a devastating prognosis. We also identified atypical cases of *PRRT2*‐positive SeLIE from those previously known to have neonatal onset or to be unremitting. These findings may expand our understanding of the phenotype and genotype of *PRRT2*‐related epilepsy and enable early patient recognition. Furthermore, we suggest the administration of SCB for patients with a distinct phenotype of *PRRT2*‐related epilepsy at the initial presentation.

## AUTHOR CONTRIBUTIONS

JHL and BCL conceived and designed the study. JWL, BCL, and YOK collected the participants' data. JWL and BCL analyzed the clinical data. JWL drafted the manuscript. JHL and BCL reviewed the data analysis and the manuscript.

## CONFLICT OF INTEREST

None of the authors has any conflict of interest to disclose.

## ETHICAL APPROVAL

This study was approved by the Institutional Review Board of Samsung Seoul Hospital (IRB No. 2022‐03‐063). The need for informed consent was waived because this study analyzed retrospective data. We confirm that we have read the journal's position on issues involved in ethical publication and affirm that this report is consistent with those guidelines.

## Supporting information


Table S1.
